# Relationship of Inflammatory Biomarkers with Severity of Peripheral Arterial Disease

**DOI:** 10.1155/2016/6015701

**Published:** 2016-07-31

**Authors:** Kimihiro Igari, Toshifumi Kudo, Takahiro Toyofuku, Yoshinori Inoue

**Affiliations:** Division of Vascular and Endovascular Surgery, Department of Surgery, Tokyo Medical and Dental University, 1-5-45 Yushima, Bunkyo-ku, Tokyo 113-8519, Japan

## Abstract

*Objective*. The pentraxin family, including high-sensitivity C-reactive protein (hs-CRP), serum amyloid P (SAP), and pentraxin 3 (PTX3), has been identified as playing a key role in inflammatory reactions such as in atherosclerosis and cardiovascular disease. In this study, we examined the relationship between peripheral arterial disease (PAD) and serum levels of pentraxins.* Methods*. This study was undertaken via a retrospective review of PAD patients with surgical intervention for lesions of the common femoral artery. We evaluated the preoperative patient conditions, hemodynamic status, such as ankle brachial index (ABI), and clinical ischemic conditions according to Rutherford classification. Preoperatively, we collected blood samples for determining the serum levels of hs-CRP, SAP, and PTX3.* Results*. Twelve PAD patients with common femoral arterial lesions were treated and examined. The hemodynamic severity of PAD was not negatively correlated with hs-CRP, SAP, or PTX3. The clinical severity evaluated by Rutherford classification was significantly positively correlated with the serum level of PTX3 (*p* = 0.019).* Conclusion*. We demonstrated that PTX3 might be a better marker of PAD than hs-CRP and SAP. Furthermore, PTX3 might be a prognostic marker to evaluate the severity of PAD.

## 1. Introduction

Inflammation is currently recognized as a critical factor involved in the development, progression, and rupture of atherosclerotic plaques and thrombus formation [1–3]. These findings prompted assessments to improve risk stratification for cardiovascular disease (CVD), which has led to the evaluation of several markers of inflammation as potential tools for CVD risk prediction. Among the most actively studied biomarkers is high-sensitivity C-reactive protein (CRP). The association between moderately elevated high-sensitivity CRP (hs-CRP) level and an increased risk for the development of cardiovascular events is well established [4].

CRP and serum amyloid P component (SAP) are classified into the pentraxin family of proteins. Pentraxins are a superfamily of proteins, phylogenetically conserved from arachnids to mammals and characterized by the presence in their carboxy-terminal of a 200-amino-acid pentraxin domain [5, 6]. CRP and SAP are referred to as short pentraxins. Pentraxin 3 (PTX3) is classified as a long pentraxin and is structurally related to but distinct from the short pentraxins [7].

In the cardiovascular field, pentraxins, including CRP, SAP, and PTX3, are well established to be useful for diagnosing CVD and indicate the disease state and disease rate [8]. However, in the field of peripheral arterial disease (PAD), few studies of the association between PAD and pentraxins have been reported [9]. Considerable evidence on the significant contribution of inflammation to the initiation and progression of atherosclerosis has been gathered over many years and there is abundance of experimental and clinical studies that support this concept. However, they have not translated into clinical practice. Furthermore, no reports have been published concerning the relationship between the levels of pentraxins and the severity of PAD. The purpose of this study was thus to compare the severity of PAD and the levels of biomarkers including hs-CRP, SAP, and PTX3 and to evaluate the efficacy of determining the levels of biomarkers for assessment of the severity of PAD.

## 2. Patients and Methods

### 2.1. Patient Selection

Between November 2012 and August 2013, 89 patients underwent 106 revascularization procedures for PAD. In this study, we included the patients who were treated by thromboendarterectomy of the common femoral artery. We hypothesized that the patient group might reveal more sensitive association between biomarkers and the severity of PAD, because the severity of femoral atherosclerosis could predict the presence of and extent of coronal calcification, a sign of coronary atherosclerosis [10], which has shown the significant correlation with pentraxins. We have to omit the influence of severe coronary atherosclerosis for accurate evaluation of the relationship between pentraxins and PAD; then we excluded the patients with unstable conditions such as acute coronary syndrome and severe heart failure. Furthermore, we excluded patients with inflammatory diseases such as collagen disease, malignancy, and infectious disease. These unstable conditions and inflammation could affect the levels of biomarkers. Finally, we included and investigated 12 patients in this study. All protocols, surveys, and consent forms were approved by the Institutional Review Board of Tokyo Medical and Dental University Hospital. Written informed consent was obtained from all subjects.

### 2.2. Clinical Data

We investigated the patients' demographics, medications, and medical histories using a questionnaire. The patients' medical records were reviewed as described below. Hypertension was defined as systolic blood pressure of >130 mmHg, diastolic blood pressure of >80 mmHg, or a history of treatment for hypertension. Dyslipidemia was defined as a serum LDL cholesterol level of >140 mg/dL, an HDL cholesterol level of <40 mg/dL, a triglyceride level of >150 mg/dL, or a history of treatment for dyslipidemia. Coronary artery disease was defined as the presence of angina pectoris, myocardial infarction, or both, as documented on coronary angiography or based on a history of any revascularization procedures of the coronary arteries. Cerebrovascular disease was defined as a history of stroke, transient ischemic attacks, carotid artery revascularization, or cerebral hemorrhage. Diabetes mellitus was defined as having fasting blood glucose ≧ 126 mg/dL or hemoglobin A1c ≧ 6.5% or being medicated with antidiabetes drugs.

### 2.3. Evaluation of PAD

We diagnosed PAD using computed tomography and/or magnetic resonance imaging and/or duplex ultrasonography and/or digital subtraction angiography. All affected limbs were preoperatively assessed using the measurement of ankle brachial index (ABI) to evaluate the severity of PAD. The patients rested in a supine position for at least 10 minutes prior to the measurements. Limb and room temperatures were kept stable at adequate levels. Systolic blood pressure was measured in both the right and the left brachial, dorsalis pedis, and posterior tibial arteries, unless contraindicated. The ABI was calculated using the standard definition, that is, the highest ankle pressure for each leg divided by the highest brachial pressure. Furthermore, the clinical severity of PAD was assessed according to Rutherford classification [11], which was categorized by the clinical features and symptoms.

### 2.4. Measurements of Blood Samples

Venous blood was drawn by vein puncture with ethylenediaminetetraacetic acid anticoagulant in the morning after overnight fast before revascularization procedures. These samples were centrifuged at 3000 rpm for five minutes, and the supernatants were separated and stored at −80°C until analysis. Laboratory analyses of the measurements for the serum levels of hs-CRP, SAP, and PTX3 were performed by SRL Inc. (Tokyo, Japan).

### 2.5. Statistical Analysis

Continuous variables are expressed as the mean and standard deviation, and categorical variables are expressed as frequencies and percentages. Statistical significance was assessed using Student's *t*-test for continuous variables and Fisher's exact test or the chi-square test for categorical variables. Pearson's correlation coefficients were used to reflect the levels of relationships between variables. A *p* value of <0.05 was considered to be statistically significant, and the statistical analyses were performed using the StatView version 5 software program (Abacus Concept Inc., Berkeley, CA, USA).

## 3. Results

### 3.1. Patient Characteristics (Table 1)

During the study period, we treated and examined 12 patients. We performed 12 thromboendarterectomies at the common femoral artery. Simultaneously, two cases of femoropopliteal bypass surgery were performed, and five cases of endovascular treatment of aortoiliac stenotic lesions and one case of endovascular treatment of femoropopliteal stenotic lesions were also carried out. The mean age of the patients was 68.6 ± 7.5 years (range: 57–88 years), and seven patients (66.7%) were male. The documented comorbidities were hypertension (100%), diabetes mellitus (75%), dyslipidemia (66.7%), coronary artery disease (33.3%), chronic kidney disease requiring hemodialysis (16.7%), and cerebrovascular disease (8.3%). The median ABI was 0.62 (range: 0.15–1.5). A few limbs had severe medial calcification of the tibial arteries, as confirmed by X-ray examinations; therefore, some patients revealed extremely high ABI (>1.3). Eight limbs were categorized as Rutherford classification level 3, three limbs were categorized as level 4, and one limb was categorized as level 5.

### 3.2. The Association between PAD Parameters and Serum Biomarkers

The mean serum biomarkers including hs-CRP, SAP, and PTX3 are shown in Table 1. In this study, we assessed the association between such biomarkers and the severity of PAD.

First, we compared hemodynamic and clinical parameters of PAD including ABI and Rutherford classification with patient comorbidities (Table 2). Mean ABI levels were significantly higher in the patients with hemodialysis and patients without cerebrovascular disease (*p* values < 0.01 and < 0.05, resp.); we consider that these patient groups included patients with extremely high ABI, so these differences might occur. Concerning Rutherford classification, we divided the categories into two groups: classification level 3 as the noncritical limb ischemia (non-CLI) group and classification levels 4 and 5 as the CLI group. In each group, there were no remarkable differences between the patients with and without comorbidities.

Second, we compared the levels of biomarkers with PAD parameters, such as the ABI levels and Rutherford classification (Table 3). Even though ABI levels might not be reliable in this study, Pearson's correlation coefficient test revealed that there were statistically significant correlations between ABI levels and hs-CRP and between ABI levels and SAP (*r* = 0.853 and *p* < 0.01 and *r* = 0.663 and *p* = 0.037, resp.). Unfortunately, these correlations revealed positive relationships between the ABI levels and the serum levels of biomarkers, which led to a discrepancy between inflammation and the severity of atherosclerosis. On the other hand, clinical evaluation of PAD status with Rutherford classification was more reliable in this study, and we compared Rutherford classification with biomarkers, which showed that PTX3 was significantly correlated with Rutherford classification (*r* = 0.661 and *p* = 0.019); PTX3 level was higher in patients with CLI than in those without it. This result is reasonable to explain the relationship between inflammation and atherosclerosis.

## 4. Discussion

The pentraxins form a superfamily of multifunctional proteins that have been phylogenetically conserved from arachnids to mammals. The pentraxin superfamily is divided structurally into two groups: the short constituents and their long counterparts [12]. CRP, a member of the short pentraxin family, is the most well-established marker of inflammation and could provide independent prognostic information for various types of CVD [13]. SAP is also a major member of the short pentraxin family [14]. CRP and SAP are acute-phase proteins, and they are synthesized in the liver under the guidance of inflammatory cytokines, most prominently interleukin-6 (IL-6) and, to a lesser degree, IL-1*β* [15]. PTX3, a member of the long pentraxin family, is rapidly induced in various cell subsets, including myeloid dendritic cells, leukocytes, and vascular endothelium, under stimulation with inflammatory cytokines, especially IL-1 and tumor necrosis factor (TNF) [16, 17]. PTX3 is synthesized locally, in the vascular system [18]; therefore, PTX3 is structurally related to, but distinct from, CRP and SAP.

In this study, PTX3 was significantly correlated with the clinical severity of PAD, and the serum level of SAP was relatively correlated with clinical symptoms (*r* = 0.565 and *p* = 0.055). These findings might reveal that peripheral atherosclerosis affects the serum levels of PTX3 and SAP. PTX3 is reported to be localized to atherosclerotic plaques [19] and to play a role in the modulation of endothelial cell procoagulant activity [20]. The PTX3 level might serve as an early indicator of myocardial infarction [21] and thus reflects the atherosclerotic activity [7]. SAP has been recognized in atherosclerotic lesions, and it is supposed to be localized with apolipoprotein in human atheroma [22]. Furthermore, the SAP levels have been revealed to be positively associated with CVD [23]. In this study, hs-CRP was not positively correlated with the severity of PAD. These results seemed to be related to PTX3 being abundantly produced by various cells in atherosclerotic lesions, whereas CRP is mainly produced in the liver [5]. These findings suggest that PTX3 levels reflect local inflammation at atherosclerotic lesions more accurately than does CRP. Similar to these findings, Katakami et al. [24] reported that PTX3 levels in type 1 diabetic patients were more closely associated with the progression of atherosclerosis than hs-CRP levels. These findings are compatible with our results in this study. hs-CRP has been shown to vary with many conditions, such as smoking, obesity, and age, and is strongly linked to a proinflammatory state [25]. However, PTX3 is independent from such states, unlike hs-CRP [7]. Therefore, PTX3 may reflect the inflammatory status of the vasculature more directly than hs-CRP.

In our study, PTX3 was found to be more strongly related to the severity of PAD than SAP. As is well known, PTX3 is produced locally in atherosclerotic lesions themselves and expressed more in peripheral tissues, with evidence of the upregulation of its level in atherosclerotic plaques [26]. Even though SAP is also produced in atherosclerotic lesions, it is mainly produced by the liver [27]. In this study, we performed thromboendarterectomy for common femoral arterial lesions; however, we could not calculate the total level of calcified plaques. Shindo et al. [28] reported that PTX3 may become a new powerful predictor of plaque vulnerability. In other kinds of CVD, especially in coronary artery disease, Peri et al. [21] reported that individual levels of PTX3 were attained independently of the extent of myocardial necrosis or incident heart failure assessed by the Killip classification. Inoue et al. [7] reported that plasma PTX3 level was significantly higher in patients with unstable angina pectoris than in normal subjects. These findings might also apply to PAD, which is one of the atherosclerotic diseases, the same as CVD.

PTX3 has been recognized as a reliable prognostic marker of not only CVD [29] but also sepsis, infectious disease, and a shock state [30–32], in all of which a significant correlation has been found between PTX3 plasma levels and disease severity. In patients with acute coronary syndrome (ACS), several biochemical markers, especially creatine phosphokinase (CPK) and troponin T, are essential in the diagnosis and prognosis of patients. However, theoretically, patients with unstable angina pectoris do not have any cardiac necrosis, and neither their CPK nor their troponin T levels are elevated. Furthermore, some studies have suggested that 5%–10% of patients with chest pain and a normal electrocardiogram will subsequently be diagnosed with unstable angina [7]. Therefore, in special cases, PTX3 could be a reliable prognostic marker of ACS. In our study, PTX3 was significantly correlated with Rutherford classification, which reflects the clinical status of PAD. We generally evaluate the severity of PAD by ABI measurement. However, ABI was not always reliable value in special cases with media calcification [33]. Therefore, PTX3 might be a more reliable prognostic marker to estimate the severity of PAD, including the cases with media calcification.

Our study has several limitations. First, the number of study subjects was quite small. Therefore, to establish the utility of the measurement of PTX3 levels, another study with a larger sample size should be performed. Second, the enrolled patients were limited to those with an arterial lesion in the common femoral artery. Furthermore, other kinds of inflammatory conditions, such as sleep apnea [34] and cardiac valvular disease [35], have shown the significant correlations with PTX3, and we could not exclude all the conditions, which might affect our result. The numbers and sites of atherosclerotic lesions in peripheral and systemic arteries might have affected our results, although complete evaluation of atherosclerosis was impossible in this study. Therefore, future prospective studies of a large cohort are needed to address these issues.

## 5. Conclusion

Our results indicate that PTX3 level is associated with the severity of PAD, and PTX3 may reflect the inflammatory status of the vasculature more directly than CRP. Even though the ABI value is a reliable parameter to estimate the severity of PAD, in some cases with media calcification, ABI values are not reliable. In such cases, PTX3 might not be affected by media calcification and may be used to evaluate the severity of PAD.

## Figures and Tables

**Table 1:** Patient characteristics.

Parameters	
Gender (male/female) (%)	7/5 (58.3/41.7)
Age (years)	68.6 ± 7.5
Comorbidities (%)	
Hypertension	12 (100)
Dyslipidemia	8 (66.7)
Coronary artery disease	4 (33.3)
Cerebrovascular disease	1 (8.3)
Diabetes mellitus	9 (75)
Chronic kidney disease on hemodialysis	2 (16.7)
Variables of peripheral arterial disease	
ABI (median [range])	0.62 [0.15–1.5]
Rutherford classification (3/4/5) (%)	8/3/1 (66.7/25.0/8.3)
Biomarkers	
hs-CRP (ng/mL)	2670.9 ± 5273.4
SAP (*μ*g/mL)	39.6 ± 19.9
PTX3 (ng/mL)	3.85 ± 3.05

ABI, ankle brachial pressure index; hs-CRP, high-sensitivity C-reactive protein; SAP, serum amyloid P; PTX3, pentraxin 3.

**Table 2:** The comparisons between comorbidities and peripheral arterial disease.

Variables (with/without)	Mean ABI	*p* value	Rutherford classification (3 : 4 and 5)	*p* value
Diabetes mellitus	0.46/0.90	NS	6 : 3/2 : 1	NS
Dyslipidemia	0.5/0.78	NS	5 : 3/3 : 1	NS
Coronary artery disease	0.67/0.57	NS	1 : 3/7 : 1	NS
Chronic kidney disease on hemodialysis	1.14/0.48	<0.01	1 : 1/7 : 3	NS
Cerebrovascular disease	0.35/0.64	<0.05	1 : 0/7 : 4	NS

ABI, ankle brachial pressure index; NS, not significant.

**Table 3:** The correlation between the severity of peripheral arterial disease and serum biomarkers.

Variables	hs-CRP		SAP		PTX3	
	*r*	*p*	*r*	*p*	*r*	*p*
ABI	0.853	<0.01	0.663	0.037	0.296	NS
Rutherford classification	0.380	NS	0.565	NS	0.661	0.019

ABI, ankle brachial pressure index; hs-CRP, high-sensitivity C-reactive protein; SAP, serum amyloid P; PTX3, pentraxin 3; NS, not significant.
